# Development of a perfusion process for serum-free adenovirus vector herpes zoster vaccine production

**DOI:** 10.1186/s13568-022-01398-7

**Published:** 2022-05-14

**Authors:** Yang Sun, Lingling Huang, Jianqi Nie, Kai Feng, Yupeng Liu, Zhonghu Bai

**Affiliations:** 1 Institute of Microbial Engineering, School of Life Sciences, Henan University, Kaifeng, 475004 China; 2 Engineering Research Center for Applied Microbiology of Henan Province, Kaifeng, 475004 China; 3 The Key Laboratory of Industrial Biotechnology, Ministry of Education, Jiangnan University, Wuxi, 214122 China; 4 National Engineering Research Center of Cereal Fermentation and Food Biomanufacturing, Jiangnan University, Wuxi, 214122 China; 5 Jiangsu Provincial Engineering Research Center for Bioactive Product Processing, Jiangnan University, Wuxi, 214122 China

**Keywords:** Herpes zoster vaccine, Adenovirus vector, Perfusion, Serum-free, Design of experiment.

## Abstract

Herpes zoster is caused by reactivation of the varicella zoster virus (VZV). Researching and developing a herpes zoster vaccine will help to decrease the incidence of herpes zoster. To increase the bioreactor productivity, a serum-free HEK293 cell perfusion process with adenovirus vector herpes zoster (rAd-HZ) vaccine production was developed efficiently using the design of experiment (DoE) method. First, serum-free media for HEK293 cells were screened in both batch and semi-perfusion culture modes. Then, three optimal media were employed in a medium mixture design to improve cell culture performance, and the 1:1 mixture of HEK293 medium and MCD293 medium (named HM293 medium) was identified as the optimal formulation. On the basis of the HM293 medium, the relationship of critical process parameters (CPPs), including the time of infection (TOI), multiplicity of infection (MOI), pH, and critical quality attributes (CQAs) (adenovirus titer (Titer), cell-specific virus yield (CSVY), adenovirus fold expansion (Fold)) of rAd-HZ production was investigated using the DoE approach. Furthermore, the robust setpoint and design space of these CPPs were explored. Finally, the rAd-HZ production process with parameters at a robust setpoint (TOI = 7.2 × 10^6^ cells/mL, MOI = 3.7, and pH = 7.17) was successfully scaled-up to a 3-L bioreactor with an alternating tangential flow system, yielding an adenovirus titer of 3.0 × 10^10^ IFU/mL, a CSVY of 4167 IFU/cells, a Fold of 1117 at 2 days post infection (dpi). The DoE approach accelerated the development of a HEK293 serum-free medium and of a robust adenovirus production process.

## Introduction

The herpes zoster virus, which belongs to the herpes virus subfamily, is a double-stranded DNA virus. Usually, latent herpes zoster virus in the dorsal root or cranial ganglion is reactivated to cause herpes zoster with common complications of post-herpetic neuralgia and chronic pain, leading to severe and painful rashes in the elderly and affecting the quality of life of patients (Harpaz 2019; Weinberg 2007). Vaccination is still one of the most effective ways to alleviate this disease. At present, only two herpes zoster vaccines are available, a live attenuated vaccine from Merck and an inactivated vaccine from GSK (Harbecke et al. 2021; Weinberg et al. 2018). Live attenuated vaccines are very effective in producing humoral and cellular immune responses, but their use for many pathogens is associated with a certain risk in humans (Hanley 2011; Minor 2015). Although inactivated and subunit vaccines provide certain safety, a re-injection of high-dose adjuvant vaccines is often required due to the limited production of an antibody response (Nieto and Salvetti 2014). Adenovirus vector vaccines’ merits include safety, high efficiency, ease of production, and the induction of cellular and humoral immunity (Kallel and Kamen 2015; Logunov et al. 2020). Thus, the development of an adenovirus vector vaccine will provide an alternative strategy for the prevention of herpes zoster.

One of the main limitations of adenovirus vector production using HEK293 cells is that cell-specific productivity decreases with increased infected cell density (known as the “cell density effect”) (Xiang et al. 2015). To solve this problem, perfusion cultivation has been used to increase the volumetric productivity of cells cultured at a high cell density (Tapia et al. 2016). During the perfusion cultivation, cells are retained in a bioreactor, while toxic by-products or metabolites, which may affect culture homeostasis, are continuously removed. Thus, high viable cell density or a continuous cell culture process could be obtained, which could potentially increase volume productivity (Butler 2005; Schwarz et al. 2020). Apparently, changing the cell culture process from a batch mode to a perfusion mode also leads to the change of virus production process parameters, such as time of infection (TOI) and multiplicity of infection (MOI). According to the quality by design (QbD) framework, to obtain a robust adenovirus production process, a robust setpoint and design space for the process parameters should be explored using the design of experiment (DoE) approach (Zhang and Mao 2017). This strategy is available for antibodies and virus vaccines (Patel et al. 2018; Poncet et al. 2020) but has seldom been reported for adenovirus production.

Culture medium is another key factor for cell cultures and vaccine yield and quality (Li et al. 2021). In industrial applications, the optimal medium for biological products must support high cell density, high cell productivity, and reduce the manufacturing costs (Kim et al. 2021). Traditional culture media (basal media supplemented with plasma or serum) could supply sufficient nutrients to cell cultures; however, their unclear composition increases the batch-to-batch variability and the risk of virus contamination (van der Valk et al. 2004). As an alternative, serum-free media (SFM) and chemically defined media could reduce the batch-to-batch variation generated by raw materials and eliminate potential microbial contamination sources (Yao and Asayama 2017). SFM have widely been used for cell cultivation of biological products, such as recombinant therapeutic proteins and vaccines (Gelinas et al. 2019; Li et al. 2021). Although several commercial SFM for HEK293 cells are available, they differ with regards to their cell culture performance and adenovirus productivity (Shen et al. 2016). Therefore, personalized SFM should be researched and developed for industrial vaccine production.

In this paper, a perfusion process for serum-free adenovirus vector herpes zoster vaccine production was developed efficiently using the DoE approach. The mixture design was conducted to obtain an optimal medium for HEK293 cells. Then, the relationship of critical process parameters (CPPs) (TOI, MOI, and pH) and critical quality attributes (CQAs) (adenovirus titer (Titer), cell-specific virus yield (CSVY), and the adenovirus fold expansion (Fold)) of adenovirus vector herpes zoster (rAd-HZ) vaccine production was investigated in shake flasks using the DoE approach. Finally, a robust rAd-HZ production process was successfully developed and scaled-up to a 3-L bioreactor with an alternating tangential flow (ATF) perfusion system. This study could be a guideline for the development of an efficient, serum-free vaccine production process.

## Materials and methods

### Cell line, media, and virus

Suspension HEK293S cells (Invitrogen, USA), adapted to serum-free culture, were used in this study. Three laboratory-customized, ready-to-use SFM (HEK293 medium (OPM, Cat. No. JN293HEK, China), YCD293 medium (OPM, Cat. No. JN293YCD, China), and MCD293 medium (OPM, Cat. No. JN293MCD, China)) and one commercially available CD293 medium (Gibco, Cat. No. 11,913,019, USA) were used. The CD293 medium was supplemented with glutamine (Gibco, Cat. No. 25,030,149, USA) to a final concentration of 4 mM before use. The Ad.MAX™ system (SignaGen Laboratories Inc., USA) was used to generate a Ad5 vector expressing glycoprotein E of Human herpesvirus (GenBank Accession No:1487709) under the control of the CMV promoter (rAd-HZ). The rAd-HZ for infection was prepared in advance and was stored in stock aliquots (2.0 × 10^9^ IFU/mL) at − 80 °C.

### Medium screening and mixture design

Suspension HEK293S cells were pre-cultured in 250-mL shake flasks (Corning, USA) with 50 mL HEK293 medium. Each flask was inoculated at 0.3 × 10^6^ cells/mL and incubated at 37 °C, 5% CO_2_ atmosphere, and 120 rpm agitation speed. The cells were adapted to a specific medium after three cell passages in it. Medium screening experiments were conducted in shake flasks using a batch culture mode and a semi-perfusion mode. The semi-perfusion culture was performed as follows: cells cultured in a shake flask were transferred into a centrifuge tube and centrifuged at 800 g for 5 min; then, the supernatant was removed and replaced with fresh medium. Semi-perfusion was performed daily (1 RV/day) or twice a day (2 RV/day). Samples were obtained daily to determine the viable cell density, cell viability, and cell metabolism.

The mixture design experiment was generated using the MODDE software (Sartorius, Germany) with cell fold expansion as the response (Table 1).

**Table 1:** Mixture design and experimental data of the responses

No.	HEK293 medium	YCD293 medium	MCD293 medium	Fold expansion
1	1	0	0	5.2
2	0	1	0	4.0
3	0	0	1	4.9
4	1/2	1/2	0	4.2
5	1/2	0	1/2	5.9
6	0	1/2	1/2	4.1
7	2/3	1/6	1/6	5.1
8	1/6	2/3	1/6	5.4
9	1/6	1/6	2/3	5.6
10	1/3	1/3	1/3	5.5
11	1/3	1/3	1/3	5.3
12	1/3	1/3	1/3	5.4

### Optimization of the rAd-HZ production process in shake flasks

To explore a robust setpoint and design space for the rAd-HZ production process parameters of TOI, MOI, and pH, a DoE experiment was performed using the simplified Reduced Combinatorial Design recommended by the MODDE software (Table 2), with the Titer, CSVY, and Fold at 2 days post infection (dpi) as responses (Table 2). Suspension HEK293S cells were cultured in 250-mL shake flasks with 50 mL medium and an initial cell density of 0.3 × 10^6^ cells/mL. A semi-perfusion operation was started on day 4 with a perfusion rate of 1 RV/day. When a cell density (TOI) of 0.4, 0.8, 1.2, or 1.6 × 10^7^ cells/mL was reached, the cells were infected with rAd-HZ at MOI of 2, 5, 10, or 15, and the pH values were 7.0 or 7.2, respectively. The perfusion rate was changed to 2 RV/day post infection to supply sufficient nutrition for virus replication. Samples were obtained for titer determination at 2 dpi and were temporarily stored in a refrigerator at − 80 °C.

**Table 2:** Reduced combinatorial design and experimental data

No.	TOI^a^	MOI^b^	pH	Titer	CSVY^c^	Fold^d^
1	4	2	7.0	1.0E + 10	2222	1111
2	4	5	7.0	9.5E + 09	1939	388
3	4	10	7.2	1.2E + 10	2667	267
4	4	15	7.2	1.3E + 10	2453	164
5	8	2	7.2	2.1E + 10	2770	1385
6	8	5	7.2	1.9E + 10	2262	452
7	8	10	7.0	6.6E + 09	821	82
8	12	15	7.0	6.3E + 09	497	33
9	12	2	7.0	5.5E + 09	435	218
10	12	5	7.2	1.6E + 10	1393	279
11	16	10	7.0	4.0E + 09	247	25
12	16	15	7.2	9.5E + 09	590	39
13	16	2	7.2	9.5E + 09	625	313
14	4	2	7.0	8.5E + 09	1574	787
15	8	2	7.2	1.7E + 10	1833	917
16	8	10	7.0	4.6E + 09	575	58

^a^ TOI: time of infection

^b^ MOI: multiplicity of infection

^c^ CSVY: cell-specific virus yield

^d^ Fold: adenovirus fold expansion

### Cell culture in a bioreactor

A 3-L benchtop bioreactor (Sartorius, Germany) with 2 L working volume was used for cultivation of HEK293S cells. The bioreactor was inoculated with HEK293S cells at 0.3 × 10^6^ cells/mL, and the parameters were set as follows: temperature of 37 °C, pH of 7.17, stirrer speed of 120 rpm, and dissolved oxygen of 40% air saturation. Perfusion was performed using an ATF cell retention device (Repligen, USA) with a hollow fiber membrane (Repligen, USA) of 0.2 μm pore size and 0.13 m^2^ filtration area. Two BT100-2 J peristaltic pumps (Longer, China) were used to supply fresh medium and to withdraw waste medium. Perfusion was started on day 4. The ATF flow rate was set at 0.7 L/min, whereas the perfusion rate was set at 1 RV/day and then changed to 2 RV/day at 12 h before the rAd-HZ infection. Samples were obtained daily to determine the viable cell density, cell viability, cell metabolism, and virus titer.

### Analytical methods

Cell density was measured using the trypan blue staining method. The concentrations of glucose, glutamine, ammonium, and lactate were measured using the BioProfile Flex2 Analyzer (Nova Biomedical, USA). The adenovirus titer was detected using the QuickTiter™ Adenovirus Titer Immunoassay Kit (Cell BioLabs, USA) and expressed as infectious units (IFU).

The data of the mixture design experiment and DoE experiment were analyzed using the MODDE software (Sartorius, Germany). Analysis of variance (ANOVA) test, used for statistical analysis, was calculated using MODDE software as well. The CSVY was the ratio of the adenovirus titer and viable cell density at the TOI. Fold was calculated by the total infectious adenovirus yielded in the culture vessel (shake flask or bioreactor) divided by the total infectious adenovirus added for the infection.

## Results

### Effects of the medium on HEK293S cell growth stability

**Fig. 1:**
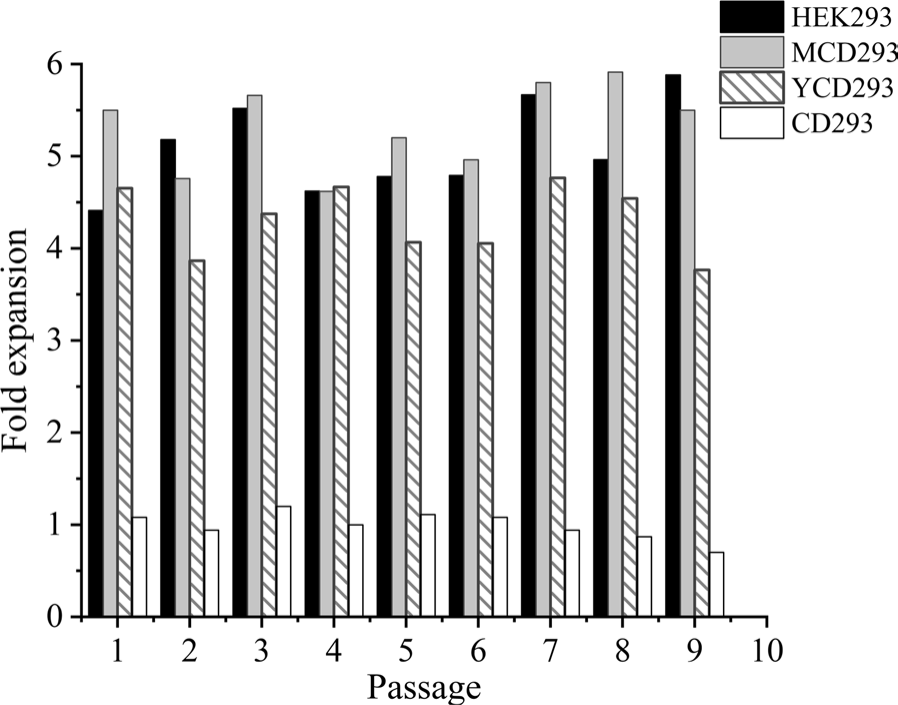
Cell fold expansion of HEK293S cells in different medium within 3 days

To evaluate the adaptability of HEK293S cells, they were grown in four SFM (HEK293, MCD293, YCD293, and CD293 medium) for 3 days and then continuously passaged nine times. As shown in (Fig. 1), the HEK293 medium and MCD293 medium performed better with regards to HEK293S cell cultivation with higher than 5-fold expansion within 3 days. Considerable fold expansion (higher than 4) was also yielded by the YCD293 medium, although it was slightly lower than that yielded by the HEK293 and MCD293 media. HEK293S cells easily achieved stable growth in those media using a direct adaptation strategy. The CD293 medium was not suitable for HEK293S cell growth, with no cell expansion within 3 days.

### Effects of the medium on HEK293S cell growth characteristics

**Fig. 2:**
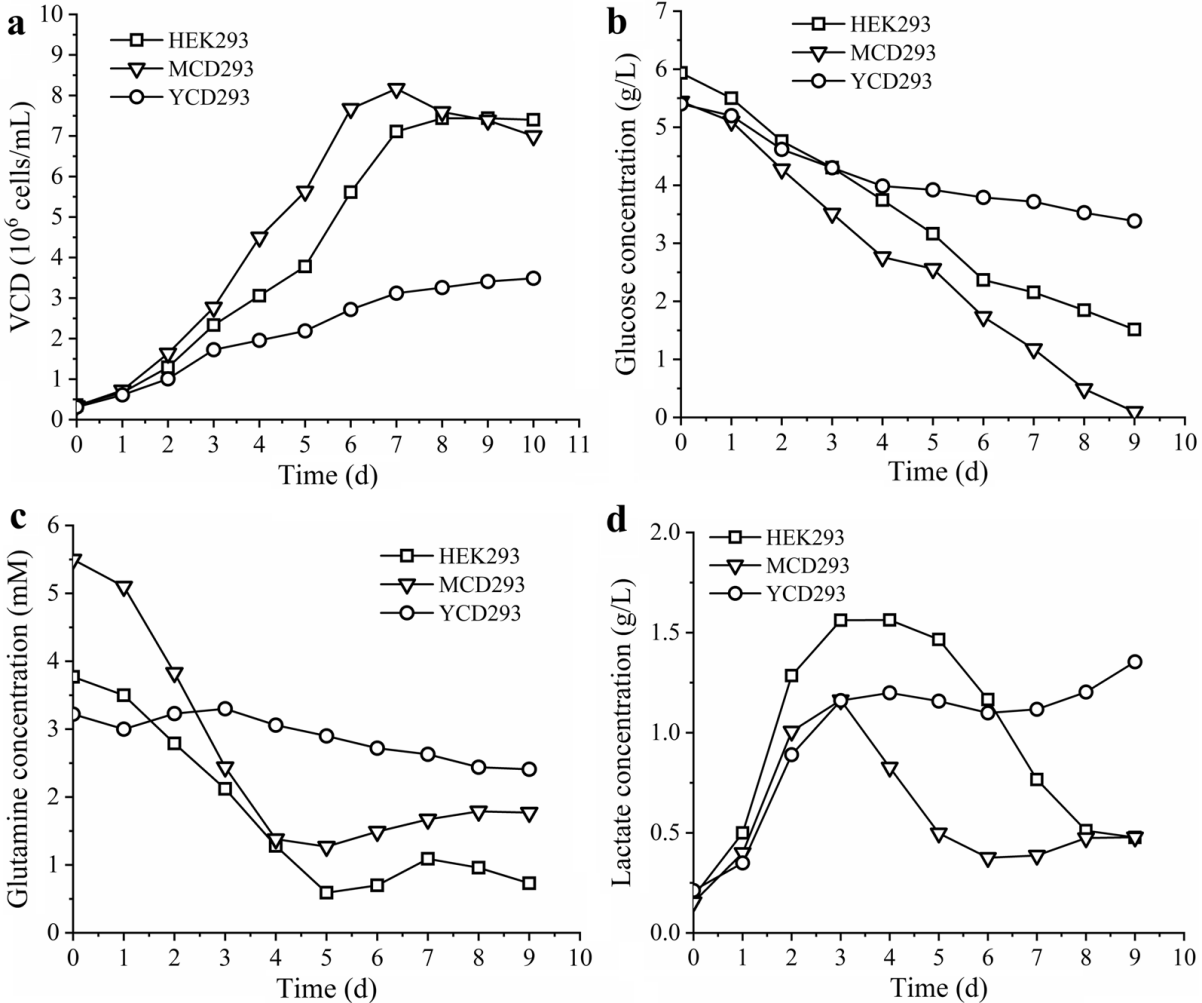
The HEK293S cells growth characteristics in batch culture mode. The cell growth curve (**a**) and metabolite profiles of glucose (**b**), glutamine (**c**), lactate (**d**)

HEK293S cell growth performance was further investigated in a batch culture mode and a semi-perfusion culture mode. In the batch culture mode (Fig. 2a), the HEK293 and MCD293 media yielded a high cell density of 7.4 × 10^6^ cells/mL (day 9) and 8.2 × 10^6^ cells/mL (day 7), respectively. A relatively lower growth rate was obtained when HEK293S cells were cultured in YCD293 medium with the cells turning to a stationary phase on day 7 at a cell density of 3.1 × 10^6^ cells/mL, which was 2-fold lower than that of cells cultured in MCD293 medium. When the cells were cultured in HEK293 medium at a high cell density, an obvious decrease of the glucose consumption rate was observed, resulting in glucose higher than 1.5 g/L on day 9 (Fig. 2b). A shift of the glutamine consumption rate was detected when the glutamine concentration decreased to ~ 1 mM (day 5), with a slight increase of glutamine (Fig. 2c). The lactate concentration increased during the first 3 days and then started to decrease, indicating that lactate was metabolically consumed by cells (Fig. 2d). Interestingly, in MCD293, lactate concentration was lower than 0.5 g/L even when the cell density reached 8.2 × 10^6^ cells/mL. The lower consumption of glucose and glutamine in YCD293 medium was in accordance with the low cell density.

**Fig. 3:**
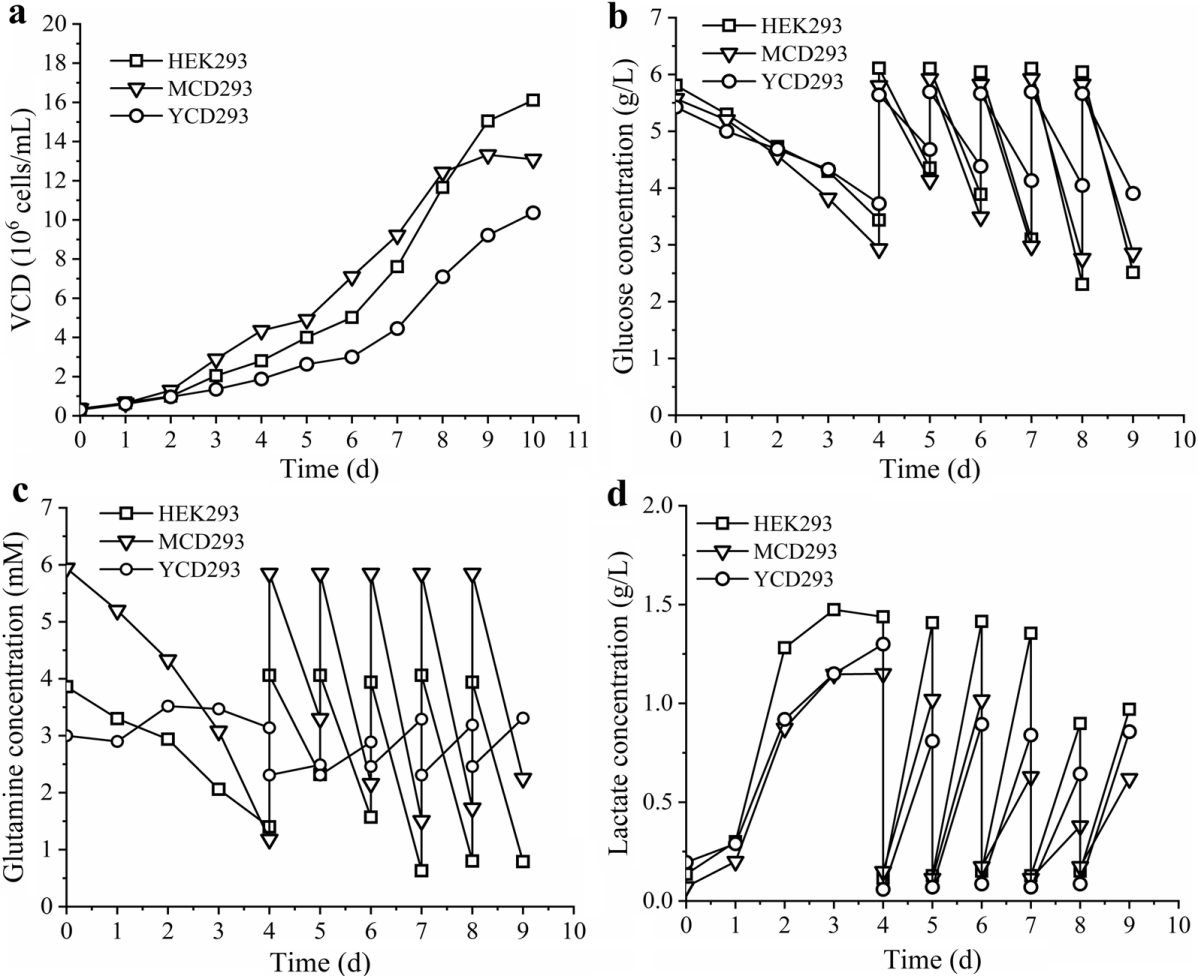
The HEK293S cells growth characteristics in semi-perfusion culture model. The cell growth curve (**a**) and metabolite profiles of glucose (**b**), glutamine (**c**), lactate (**d**)

A semi-perfusion culture of HEK293S cells was also performed in these media (HEK293, MCD293, YCD293) to investigate medium culture performance for high cell density (Fig. 3). The semi-perfusion operation was started on day 4 with a perfusion rate of 1 RV/day. Compared with the batch culture mode, the semi-perfusion strategy resulted in an ~ 2-fold increase of cell density (Figs. 2a,  3a). The HEK293 and MCD293 media yielded a comparable growth curve, with the highest cell densities being 1.6 × 10^7^ cells/mL (day 10) and 1.3 × 10^7^ cells/mL (day 9), respectively (Fig. 3a). The use of the semi-perfusion strategy also increased HEK293S cell density in the YCD293 medium, reaching 1.0 × 10^7^ cells/mL on day 10. We hypothesize that cell density was increased by elevating the supplementation with some unknown nutrients during the semi-perfusion process, with glucose concentration higher than 3 g/L and glutamine concentration higher than 2 mM (Figs. 2b, c and 3b, c). The lactate production rate was elevated during the semi-perfusion phase, while lactate was still maintained at levels lower than 1.5 g/L (Fig. 3d). Overall, the HEK293, MCD293, and YCD293 media were suitable for HEK293S cell growth at a high cell density.

### Mixture design for the HEK293S cell medium

**Fig. 4:**
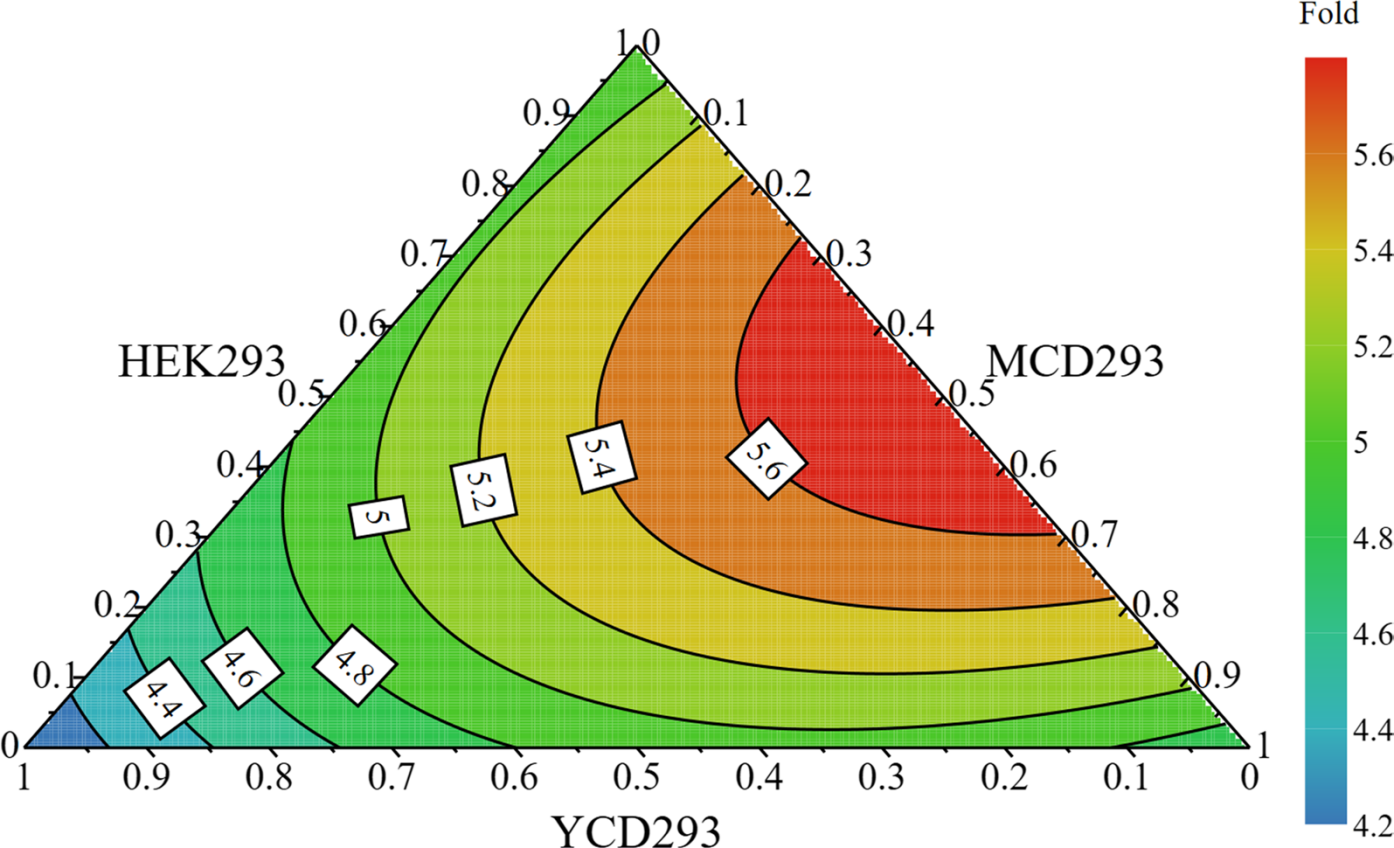
The contour plot of mixture design with cell fold expansion as response

To further improve the culture performance of HEK293S cells, HEK293, MCD293, and YCD293 media were mixed in a design experiment. The experimental matrix generated by the MODDE software is shown in Table 1. The media were mixed as demonstrated in Table 1, and HEK293S cells were passaged three times in each medium. Cell fold expansion within 3 days was used as the model response. According to the contour plot shown in Fig. 4, the mixture of HEK293 and MCD293 media further improved the cell culture performance. This may be because these two media complement each other with regards to nutrition supply. However, adding or increasing the proportion of the YCD293 medium in the mixture medium led to a decrease in cell fold expansion (Table 1). The optimal medium predicted based on the model was a mixture of HEK293 and MCD293 media at a ratio of 1:1 (named HM293 medium) with a cell fold expansion of 5.9 within 3 days, which was 1.4-fold higher than that achieved with the HEK293 medium. Thus, the HM293 medium was used in the subsequent experiments.

### Optimization of rAd-HZ production using the DoE approach

**Fig. 5:**
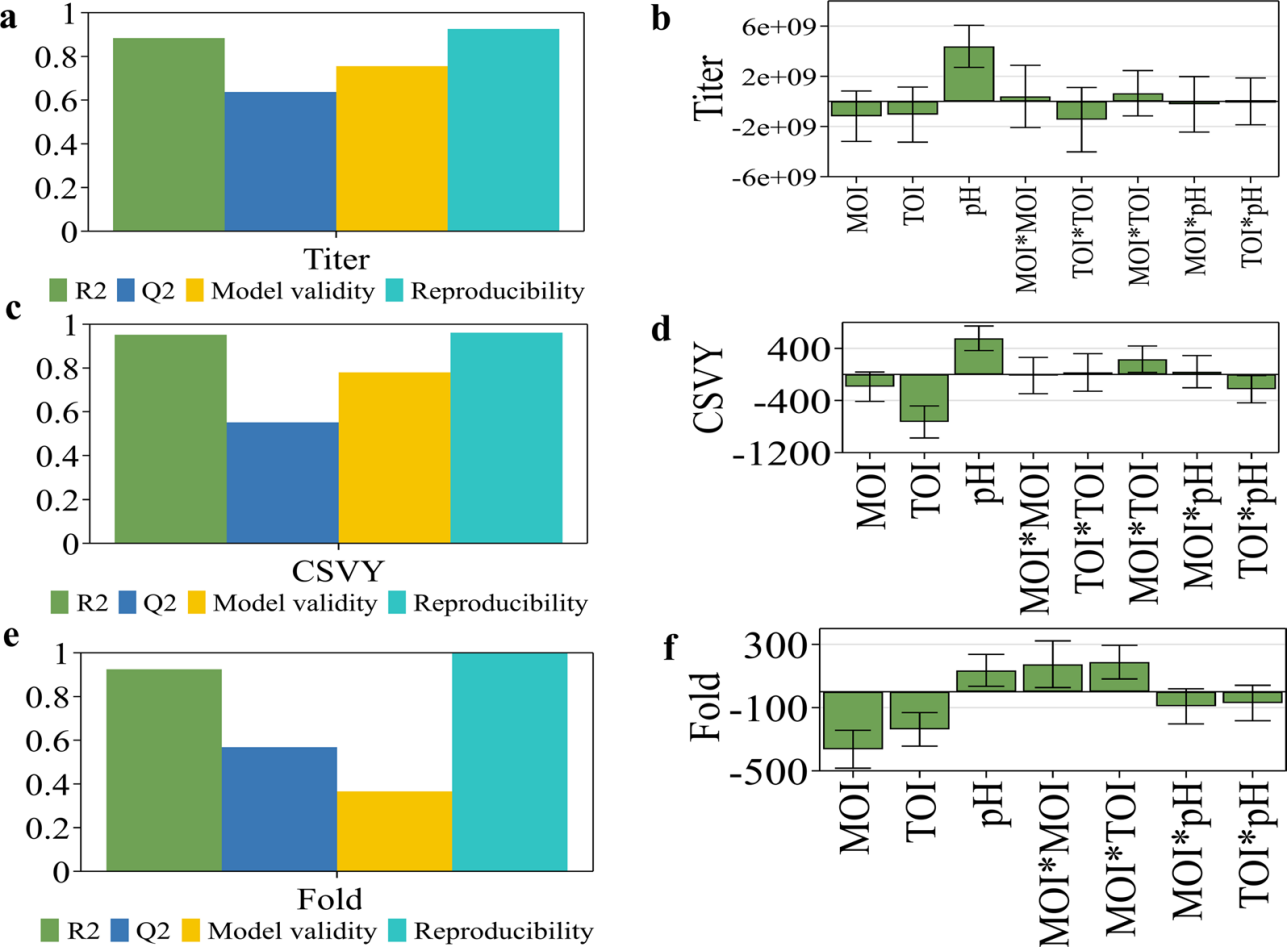
The model analysis of DoE experiment. The summary of fit and coefficients for titer (**a**, **b**), CSVY (**c**, **d**) and Fold (**e**, **f**)

To optimize the HEK293S cell-based rAd-HZ production process with HM293 medium, the CPPs of TOI, MOI, and pH were evaluated in shake flasks with Titer, CSVY, and Fold at 2 dpi as the CQAs. The internal relationship between CPPs and CQAs was investigated through fitting the DoE data (Table 2) using a multiple linear regression. The mathematical model was fitted automatically and modified manually. The model analysis is shown in Table 3; Fig. 5. The models were well fitted and had a high prediction accuracy, with R^2^ = 0.884 and Q^2^ = 0.637 for Titer, R^2^ = 0.951 and Q^2^ = 0.551 for CSVY, and R^2^ = 0.925 and Q^2^ = 0.568 for Fold. pH had significant negative effects for Titer, and CSVY, Fold, indicating that high pH may be suitable for rAd-HZ production.

**Table 3:** Coefficients and ANOVA results for the reduced combinatorial model

Terms^a^	Titer		CSVY^b^		Fold^c^	
	Coefficient	P value	Coefficient	P value	Coefficient	P value
Constant	3.13E + 11	< 0.001	− 6.51E + 04	< 0.001	− 2.69E + 04	0.02
MOI	2.44E + 09	0.21	−7.05E + 02	0.09	9.98E + 02	< 0.001
TOI	1.08E + 09	0.29	3.45E + 03	< 0.001	1.06E + 03	< 0.001
pH	4.56E + 10	< 0.001	9.75E + 03	< 0.001	4.09E + 03	0.02
MOI*MOI	1.53E + 07	0.72	− 6.52E−01	0.89	6.70E + 00	0.03
TOI*TOI	8.32E + 07	0.22	1.76E + 00	0.81	8.78E + 00	–
MOI*TOI	3.04E + 07	0.42	1.10E + 01	0.03	− 1.75E + 02	< 0.001
MOI*pH	4.46E + 08	0.81	8.08E + 01	0.70	− 1.66E + 02	0.09
TOI*pH	2.73E + 06	1.00	− 5.27E + 02	0.04	− 2.69E + 04	0.18
Regression	–	0.01	–	0.00	–	0.00
Lack of fit	–	0.38	–	0.42	–	0.08

^a^ TOI: time of infection, MOI: multiplicity of infection

^b^ CSVY: cell-specific virus yield

^c^ Fold: adenovirus fold expansion

The robust setpoint and design space were explored according to the criteria of a rAd-HZ titer higher than 1.2 × 10^10^ IFU/mL, CSVY higher than 1500 IFU/cell, Fold greater than 400, and failure probability less than 3%. After 5000 Monte Carlo simulations were carried out using the MODDE software, the robust setpoint and design space were obtained. The generated robust setpoint was a TOI of 7.2 × 10^6^ cells/mL, an MOI of 3.7, and a pH of 7.17. Under these conditions in the HM293 medium, the predicted rAd-HZ titer at 2 dpi could reach 1.6 × 10^10^ IFU/mL, and the experimental rAd-HZ titer was 2.1 × 10^10^ IFU/mL, which was 1.6-fold higher than that in the HEK293 medium (1.3 × 10^10^ IFU/mL, data not shown), MCD293 medium (1.0 × 10^10^ IFU/mL, data not shown) and YCD293 medium (9.6 × 10^9^ IFU/mL, data not shown). The design space hypercube was a TOI of 6.4–8.0 × 10^6^ cells/mL, an MOI of 2.0–4.6, and a culture pH of 7.16–7.20.

### Process validation in a bioreactor

**Fig. 6:**
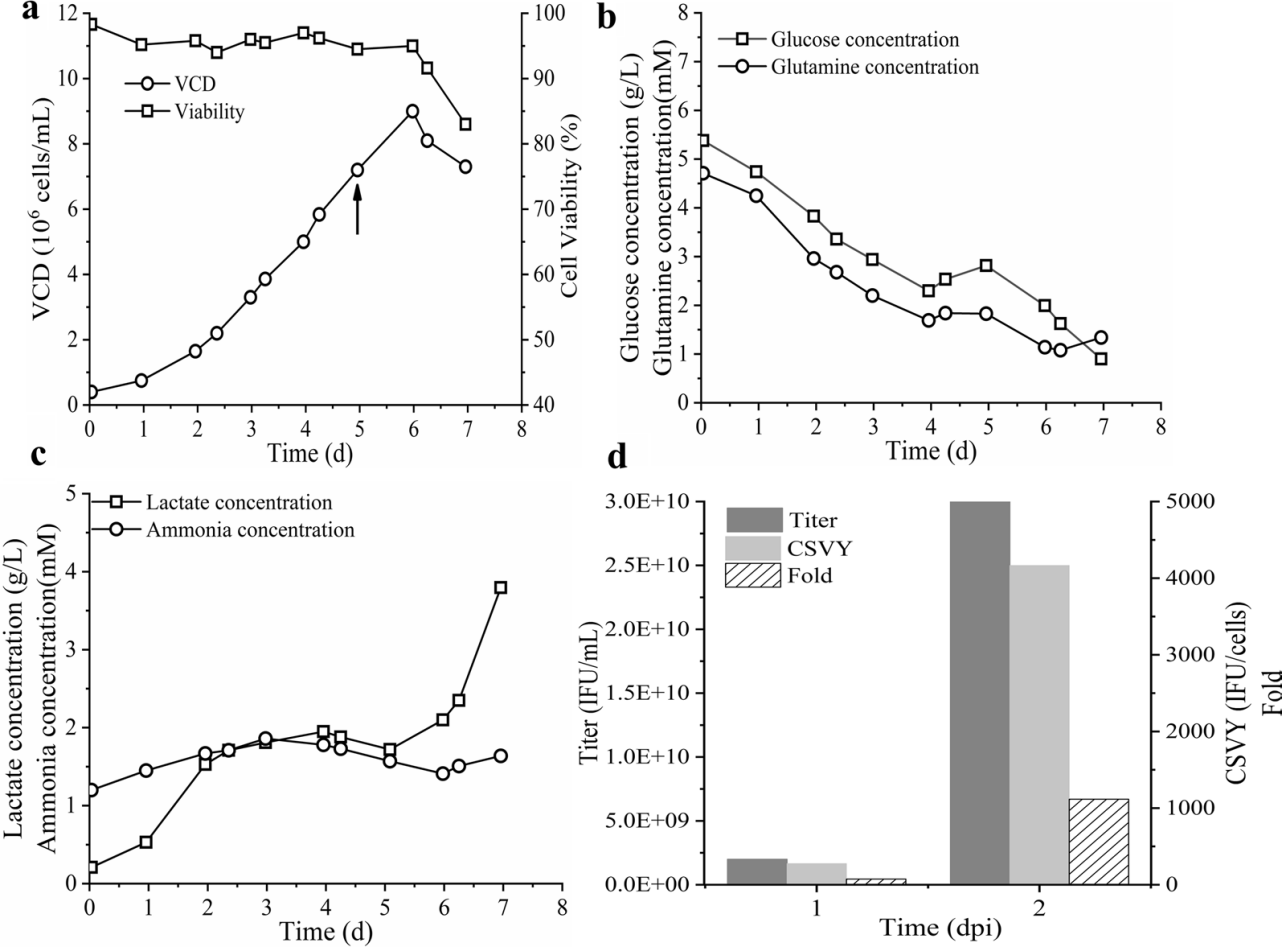
rAd-HZ production in a bioreactor with process parameters under robust setpoint conditions. A cell growth curve (**a**), metabolic curves (**b**, **c**), and rAd-HZ yield (**d**). The arrow indicates the time of infection

The rAd-HZ production process under robust setpoint conditions was scaled up to a 3-L bioreactor connected with the ATF system. The cell density reached 7.2 × 10^6^ cells/mL at day 5, and the cells were then infected with the rAd-HZ at an MOI of 3.7. After the infection (Fig. 6a), the cell density showed a trend toward an increase, followed by a decrease to 7.3 × 10^6^ cells/mL on day 7 (2 dpi). Cell viability was higher than 90% during the cell culture process; it started to decrease after the infection and reached 83% on 2 dpi. The metabolic curves are shown in Fig. 6b, c. Although an obvious increase of glucose consumption was observed after the infection, glucose could be maintained higher than 0.9 g/L through perfusion cultivation. Glutamine was maintained higher than 1 mM during the whole process. Lactate increased sharply after the infection and reached 3.8 g/L on day 7 (2 dpi). Ammonium was maintained relatively stable at 1.5 mM during the whole culture process. In the bioreactor with a perfusion system, a rAd-HZ titer of 3.0 × 10^10^ IFU/mL was obtained on day 7 (2 dpi), with a CSVY of 4166 IFU/cells and a Fold of 1117 (Fig. 6d).

## Discussion

The herpes zoster vaccine can effectively reduce the incidence and severity of herpes zoster in individuals aged at least 60 years, ensuring their quality of life (Lal et al. 2015). Prevention may be the key to reduce the disease and economic costs of herpes zoster (Jeon 2015). Adenovirus vectors are characterized by a high transduction efficiency, a high titer, low cost, a rapid response, and rapid production, rendering them potential vaccine vectors for emerging infectious diseases (Sakurai et al. 2022; Xiang et al. 2015). Currently, approved adenovirus vector vaccines include the Ebola vaccine (Majhen et al. 2014) and COVID-19 vaccine (Zhu et al. 2020).

The use of SFM for the production of human vaccines and therapeutic proteins is desirable, because the use of serum has many drawbacks, such as increasing the risk of contamination with infectious viruses and mycoplasma and considerable lot-to-lot variability caused by quantitative and qualitative serum variations (Rodrigues et al. 2013; Toriniwa and Komiya 2007). However, commercial SFM may fail to meet the personalized nutrition needs of specific cell lines or biological products. To date, various methods have been proposed to optimize cell culture media, such as metabolism (Stoll et al. 1996), consumption analysis, and mixture design (Jordan et al. 2013; Rouiller et al. 2013). As a specific application of the DoE method, mixture design could further improve medium performance by mixing several commercial or self-made SFM that complement each other with regards to nutrition supply. Due to its scientific basis and efficiency, mixture design is widely used in the development of SFM or special culture media for specific cell lines (Jordan et al. 2013). In this study, four SFM showed different culture performance, such as cell-specific growth rates and metabolic pathways. Using the mixture design, the HM293 medium (a mixture of HEK293 and MCD293 media at a ratio of 1:1) was obtained, and it showed an obvious increase in HEK293S cell culture performance and adenovirus productivity. This result indicated these two media complement each other with regards to nutrition supply. Though the composition of these medium is undisclosed, the approach in this study could help the biological products manufacturer obtain a customized medium with better performance from medium supplier.

During the HEK293 cell culture process, glucose is the main source of energy and carbon. Glutamine is an important precursor for the synthesis of proteins and nucleotides and a significant respiratory fuel for rapidly dividing cells and cells with inefficient glucose use (Lin et al. 2017). The metabolism of glucose and glutamine leads to the accumulation of lactate and ammonium, which inhibit cell growth (Jordan et al. 2013; Pereira et al. 2018). Reducing these by-products has been one of the main goals in the past few years (Mulukutla et al. 2012). To overcome nutrition limitations and metabolic waste inhibition at high cell density, the use of a perfusion strategy with cell retention devices and continuous medium refreshment is now a new trend in adenovirus manufacturing, which could yield high cell density and biological productivity (Kamen and Henry 2004). Among the commercially available perfusion systems, the ATF system driven by a diaphragm pump could provide a low shear device and potentially improve process performance. Thus, the ATF system has been widely used for the production of vaccines, such as the influenza vaccine (Wu et al. 2021). In this paper, the ATF system yielded a cell density of 7.2 × 10^6^ cells/mL with sufficient nutrition and low metabolic waste accumulation. The cell-specific virus yielded at 2 dpi was 4167 IFU/cells, indicating no “cell density effect” at such cell density.

The QbD approach, which emphasizes the product and process understanding, design space, control strategies, and continual improvement strategy, has already been widely applied in the pharmaceutical industry (Pramod et al. 2016). Thus, the herpes zoster vaccine production process should be developed using the QbD approach while also following the requirements of the International Conference on Harmonization (ICH) Q8 guidance document. Based on previously accumulated data and experience, we initiated a serum-free rAd-HZ production process development using the DoE approach, an efficient tool of the QbD strategy. To shorten the research and development cycle of a candidate vaccine, an efficient platform should be established. The DoE approach, which could reveal the interaction between variables and optimize multiple variables at multiple levels, is an efficient tool for the optimization of process parameters. In this study, the DoE approach combined with shake flasks was used as an efficient platform for the optimization of media and rAd-HZ production process parameters. The coefficient results (Fig. 5) indicated that the TOI, MOI, and pH parameters selected in this study were CPPs. Among them, pH had the most significant effect on virus replication with an optimal pH value of 7.17. The MOI could affect the growth and metabolism of infected cells and the efficiency of adenovirus amplification. The increase of MOI enhanced the cell death rate and promoted the formation of defective interfering particles (DIPs) (Frensing 2015). Finally, the robust setpoint and design space of CPPs were explored, and the changed parameters in the multidimensional space could guarantee good CQAs and were not considered a process change.

This study demonstrated the successful application of the DoE approach in the optimization of the rAd-HZ production process using a serum-free medium. Four SFM were selected for medium screening. Then, three outstanding media were selected for mixture design, and the optimal mixed medium of HM293 (a 1:1 mixture of HEK293 and MCD293 media) was used for rAd-HZ production. Using the DoE approach, the rAd-HZ production process was optimized efficiently, and the design space and robust setpoint were also obtained. Finally, the rAd-HZ production process with parameters at a robust setpoint was scaled up to a 3-L bioreactor with an ATF perfusion system, which yielded a cell density of 7.2 × 10^6^ cells/mL and a rAd-HZ titer of 3.0 × 10^10^ IFU/mL. This study could be used as a generic platform for the efficient development of SFM and biological products for human use.

## Data Availability

All data are presented in figures and tables within this article. Any material used in this study will be available for research purposes upon request.

## Abbreviations

VZV: Varicella zoster virus.
rAd-HZ: Adenovirus vector herpes zoster.
DoE: Design of experiment.
CPPs: Critical process parameters.
TOI: Time of infection.
MOI: Multiplicity of infection.
CQAs: Critical quality attributes.
Titer: Adenovirus titer.
CSVY: Cell-specific virus yield.
Fold: Adenovirus fold expansion.
dpi: Days post infection.
QbD: Quality by design.
SFM: Serum-free media.
ATF: Alternating tangential flow.
IFU: Infectious units.
ICH: International Conference on Harmonization.
DIPs: Defective interfering particles.
